# Comparison of ADO-II percutaneous occlusion and traditional surgery in the treatment of doubly committed subarterial ventricular septal defects in children

**DOI:** 10.3389/fcvm.2025.1541796

**Published:** 2025-04-15

**Authors:** Bei Tan, Mi Li, Kaijun Zhang, Xue Zhou, Qiuyue Ao, Dan Yin, Zhenli Cheng, Ping Xiang

**Affiliations:** ^1^ Children’s Hospital of Chongqing Medical University, Chongqing, China; ^2^ Department of Cardiovascular Medicine, Children’s Hospital of Chongqing Medical University, Chongqing, China; ^3^ National Clinical Research Center for Child Health and Disorders, Chongqing, China; ^4^ Ministry of Education Key Laboratory of Child Development and Disorders, Children’s Hospital of Chongqing Medical University, Chongqing, China; ^5^ Chongqing Key Laboratory of Pediatrics, Children’s Hospital of Chongqing Medical University, Chongqing, China; ^6^ Key Laboratory of Children’s Important Organ Development and Diseases, Chongqing Municipal Health Commission, Chongqing, China; ^7^ National Clinical Key Cardiovascular Specialty, Chongqing, China

**Keywords:** doubly committed subarterial ventricular septal defect, amplatzer duct occluder-II, interventional cardiology, children, surgical repair

## Abstract

**Objective:**

The aim of this study is to evaluate the efficacy and safety of ADO-II percutaneous occlusion and traditional open-chest surgery for treating doubly committed subarterial ventricular septal defect (dcVSD) in children.

**Methods:**

The clinical data of 151 children with dcVSD treated at Chongqing Medical University Affiliated Children's Hospital between July 2019 and May 2024 were retrospectively analyzed. Patients were divided into a transcatheter group (percutaneous occlusion) and a surgical group (open-chest repair) on the basis of the treatment method used. Key evaluation metrics included procedural success rates, complication rates, and perioperative management parameters.

**Results:**

Occlusion technical success was 94.9% (37/39) in the interventional sample of 39 patients. The 112 surgical patients had a 100% technical success rate. Three interventional patients had sinus rhythm before discharge, and 2 of 18 surgical patients had residual right bundle branch block at the last follow-up. The mild aortic valve prolapse of 115 individuals (76.2%) improved to varied degrees postoperatively. Of 96 individuals with preoperative aortic regurgitation, 83 exhibited no change, 49 improved, 17 developed new regurgitation, and two worsened. The two groups differed significantly in postoperative hospital stay, time to independent ambulation, operative time, mechanical ventilation, blooding amount, Blood transfusion volume, Fever within 72 h after operation, pulmonary infections, intravenous nutrition, antibiotic use, and hospitalization cost (all *p* < 0.05). There no serious problems were recorded the transcatheter group, including device dislodgement, cardiac or vascular perforation, death, or hemolysis. In the surgical group, one patient had residual shunting reoperation and another had infective endocarditis.

**Conclusion:**

Children with dcVSD can recover faster and safer using ADO-II percutaneous occlusion, which is minimally invasive and inexpensive. It can be the first-line treatment for selected patients.

## 1 Introduction

DcVSD constitute a relatively unique subtype of ventricular septal defects. They are anatomically characterized by “discontinuity” between the aortic media, aortic annulus, and ventricular septum, resulting in an extremely short distance between the superior margin of the defect and the aortic root (1). DcVSD are rare and often do not close spontaneously under natural conditions, representing approximately one-quarter of all ventricular septal defects requiring closure (2). Among adult patients, dcVSDs are associated with aortic sinus aneurysms (Valsalva sinus) (3). Cho et al. (4) reported that in a study of 60 untreated small dcVSD patients, 8.3% (5 patients) required emergency surgery due to rupture of a Valsalva aneurysm. Furthermore, as the disease progresses, untreated dcVSD may lead to progressive aortic valve dysfunction and even severe complications such as heart failure. Therefore, most patients require early intervention and treatment.

Currently, dcVSDs are primarily repaired via surgery under cardiopulmonary bypass. Although this procedure has a high success rate, it is highly invasive, and both cardiopulmonary bypass and open-chest surgery may lead to various postoperative complications. For children and adolescents, the psychological impact of scarring from traditional sternotomy should not be overlooked. In recent years, advancements in interventional techniques and occluder construction have led to the introduction of minimally invasive percutaneous interventional closure as a potential treatment option for VSDs, even as the first-line recommendation in select cases, with acceptable short- to midterm follow-up outcomes (5, 6). However, interventional closure of dcVSD is difficult owing to their unique anatomical structure. Although eccentric occluders and ADO-I occluders have been used in some institutions, the associated complications such as postoperative aortic regurgitation remain a source of concern (7, 8). Benefiting from advancements in material science, a flexible and soft polyester-based occluder— the Amplatzer duct occluder-II (ADO-II)—has become a focus of attention. While the ADO-II was not initially designed for VSDs, it has been widely adopted because of its high success rate in closing VSDs (particularly pmVSDs and mVSDs) and low complication rate (9).

Theoretically, softer occluders may better adapt to the shape of the dcVSD, causing less damage to the closely adjacent aortic and pulmonary valve and exerting minimal impact on cardiac conduction after implantation. The ADO-II has been successfully used for dcVSD closure in some institutions. Tang et al. (10) used the ADO-II to treat 24 selected pediatric dcVSD patients and reported successful closure in 23 cases. During a follow-up period of more than 45 months, only one case of new mild aortic regurgitation was reported, and no other complications were observed, suggesting that the technique is safe and feasible. Lin et al. (11) used the ADO-II to close outlet VSDs in 49 patients, resulting in 45 successful closures with no observed worsening of aortic regurgitation over a maximum follow-up period of 51.1 months. As opportunities and challenges coexist, our understanding of the efficacy and safety of using the ADO-II for dcVSD closure remains limited.

The aim of this study is to evaluate the efficacy and safety of ADO-II percutaneous occlusion for dcVSD in children and compare its outcomes and costs with those of traditional open surgical repair, hopefully providing a relatively less invasive treatment option for selected patients.

## 2 Research subjects and methods

### 2.1 Study design and patients

**Figure 1:**
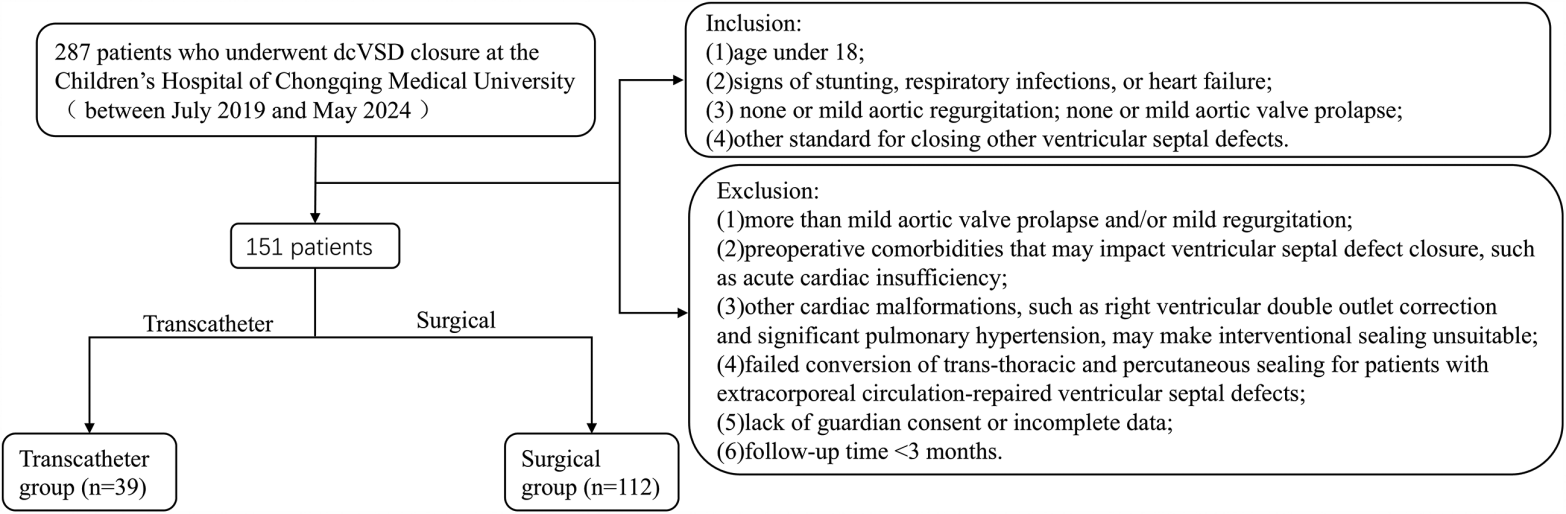
Flowchart.

This study utilized a retrospective case-control design, it was conducted on patients who underwent closure for dcVSD at the Children's Hospital of Chongqing Medical University from July 2019 to May 2024. A total of 151 patients were included in the study and divided into two groups based on the type of surgery received: the transcatheter group (*n* = 39, undergoing percutaneous closure) and the surgical group (*n* = 112, undergoing cardiopulmonary bypass repair of the VSD). Figure 1 shows a flowchart of the inclusion/exclusion of patients. Inclusion criteria were: (1) age under 18; (2) signs of stunting, respiratory infections, or heart failure; (3) none or mild aortic regurgitation; none or mild aortic valve prolapse; (4) other standard for closing other ventricular septal defects. Exclusion criteria were: (1) more than mild aortic valve prolapse and/or mild regurgitation; (2) preoperative comorbidities that may impact ventricular septal defect closure, such as acute cardiac insufficiency; (3) other cardiac malformations, such as right ventricular double outlet correction and significant pulmonary hypertension, may make interventional sealing unsuitable; (4) failed conversion of trans-thoracic and percutaneous sealing for patients with extracorporeal circulation-repaired ventricular septal defects; (5) lack of guardian consent or incomplete data;(6) follow-up time <3 months.

### 2.2 Occluder

The Amplatzer Duct Occluder (ADO-II) is a double-disc device made of nitinol wire mesh, without internal filling material, and is used for the closure of dcVSD as an “off-label use”. The devices used in this study were manufactured by AGA Medical Corporation (MN, USA) and Starway Medical Technology Inc. (Beijing, China). It needs to be mentioned is that the device has undertaken and completed phase 0 animal experiments.

### 2.3 Procedure

#### 2.3.1 Percutaneous interventional closure

**Figure 2:**
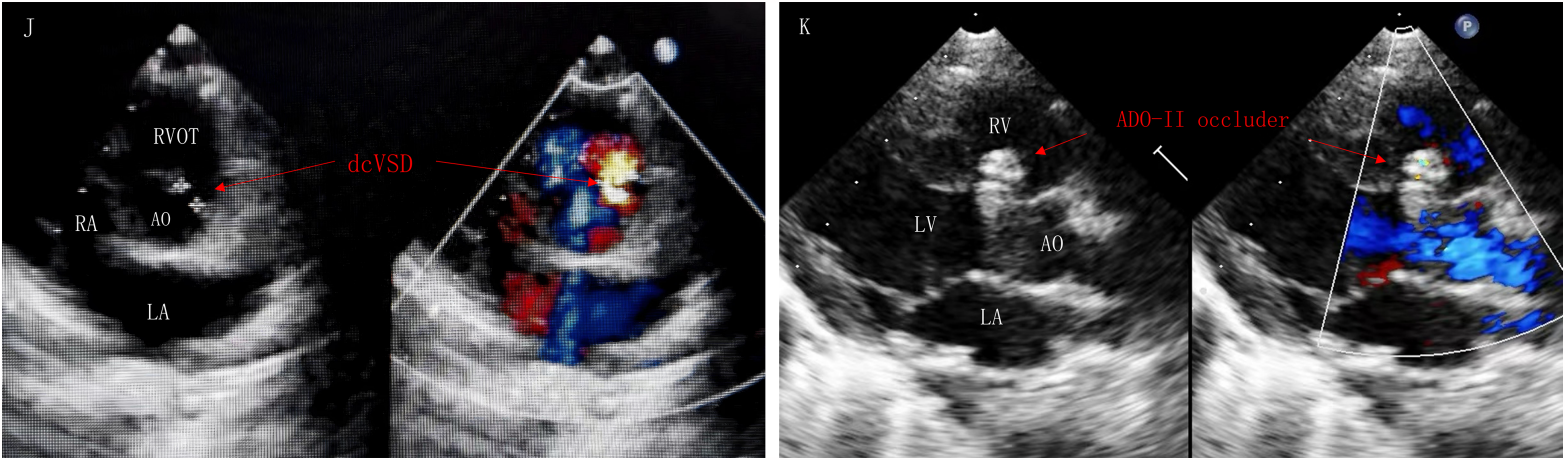
Parasternal short-axis view of the great vessels **(J)**: postoperative TTE showed dcVSD. Left sternal border long-axis view of the left ventricle **(K)**: dcVSD was successfully occluded by ADO-II occlude without AR and RS. AR, aortic regurgitation; AO, aorta; LA, left atrium; LV, left ventricle; RV, right ventricle; RS, residual shunt; RVOT, right ventricular outflow tract.

**Figure 3:**
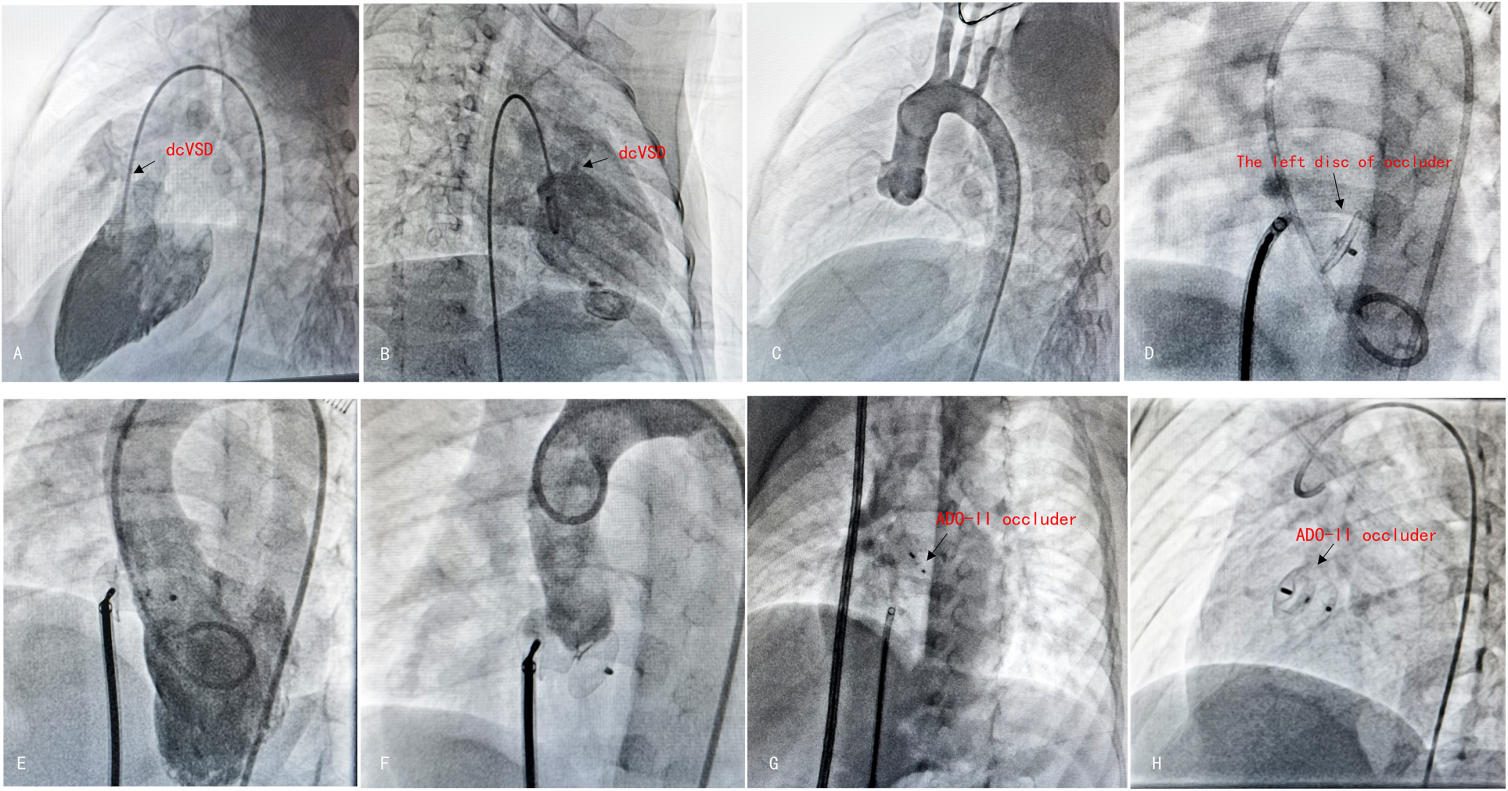
The procedure of antegrade dcVSD closure. **(A,B)** Left ventricular angiography was performed to confirm the position and size of dcVSD from two different angles—left anterior oblique at 60°cranial 20° **(A)** and right anterior oblique at 20° **(B)**. **(C)** Ascending aortogram was performed to observe for AR and AVP. **(D)** After establishing the delivery pathway, the left disc was deployed at the appropriate position. **(E,F)** Left ventricular and aortic angiography were repeated to confirm the absence of RS and no exacerbation of AR. **(G)** Released the right disc. **(H)** A final radiograph was taken to confirm the occluder's position.

The interventional group typically employed transthoracic echocardiography for continuous intraoperative cardiac monitoring. The treatment was performed under general anesthesia, a 5F/6F/7F arterial and venous sheath was placed, and heparin (100 U/kg) was injected for anticoagulation. Using a guidewire through the femoral vein, the delivery system was progressed sequentially into the right femoral vein, inferior vena cava, right atrium, right ventricle, and across the VSD into the left ventricle. Pressure curves were recorded, and blood flow was calculated. During the procedure, left ventricular and aortic angiography was conducted using at a left oblique projection at 90°/cranial tilt of 20° (or right oblique at 30°/cranial tilt of 20°) to examine the VSD shunt and the condition of the aortic valve. Under fluoroscopic guidance, the delivery sheath was pushed through the VSD into the left ventricle, and an appropriately sized occluder was selected based on preoperative ultrasonography and angiographic data. After deploying the occluder, angiography and intraoperative echocardiography were used to confirm the appropriate position of the device, absence of residual shunting, unobstructed outflow tracts of the left and right ventricles, and normal valve function. The sheath was subsequently removed, and hemostasis was established by compression. Postoperatively, dexamethasone was provided intravenously for 3–5 days (Figures 2, 3).

#### 2.3.2 Repair under cardiopulmonary bypass

After the patient was placed under combined venous anesthesia, a median sternotomy was performed, followed by layer-by-layer entry into the thoracic cavity for exploration and heparinization (about 375 U/kg, the heparin dose will be adjusted in accordance with the monitoring results to guarantee the anticoagulation effect and safety during surgery, and the coagulation function will be closely monitored). Cardiopulmonary bypass was provided, the ascending aorta was constricted, the pulmonary artery was incised transversely to fully expose and measure the edges of the dcVSD, and transesophageal echocardiography for intraoperative real-time monitoring. A Gore-Tex patch (made of expanded polytetrafluoroethylene) was correctly trimmed, and the first stitch, with a pledget, was put at the inferior-posterior margin of the dcVSD using an over-and-beyond shallow suture technique. It was repaired using a continuous suture with 5-0 Prolene, ensuring no injury to the aortic valve or conduction bundle. After sealing the incision, the patient was rewarmed, the ascending aorta was unclamped, and the heart spontaneously recovered sinus rhythm. Cardiopulmonary bypass was gradually eased off once vital signs stabilized. After assuring appropriate hemostasis, the chest was closed. Postoperatively, the patient was transported to the cardiac intensive care unit, where they received preventive antibiotics (second-generation cephalosporins), inotropic support, diuretics, sedation, and analgesics. Once the condition stabilized, the patient was successfully extubated and transferred to a general ward.

### 2.4 Follow-up

Children in the interventional group were given aspirin at a dose of 3–5 mg/kg/day for anticoagulation for six months postoperatively. For two groups, patients underwent continuous monitoring with dynamic electrocardiography (ECG), 12-lead ECG, and echocardiography preoperatively, intraoperatively in real time, on the first postoperative day, one week postoperatively, and before discharge. Comprehensive follow-up assessments, including clinical signs, ECG, and cardiac echocardiography, were conducted for both groups at 1 month, 3 months, 6 months, and 1 year after discharge, and subsequently at 1-year intervals in outpatient clinics.

### 2.5 Statistical analysis

Categorical variables are expressed as counts (percentages) and analyzed using the χ^2^ test or Fisher's exact test. Continuous variables exhibiting non-normal distributions are represented as medians (interquartile ranges). The Kolmogorov–Smirnov test was utilized to evaluate normality. Independent *t*-tests were utilized for regularly distributed data, whilst the Mann–Whitney *U*-test was employed for non-normally distributed data. All analyses were conducted utilizing IBM SPSS version 26.0 (SPSS Inc., Chicago, IL, USA). A two-tailed *p*-value of less than 0.05 was deemed statistically significant.

### 2.6 Ethics statements

The Ethics Committee of Children's Hospital of Chongqing Medical University reviewed and approved the study protocol, with an approval number of 2024-422.

## 3 Results

### 3.1 Baseline characteristics

The transcatheter group consisted of 39 patients (26 males, 13 females), ranging in age from 2 years to 15 years and 7 months, with the youngest being 2 years old (11 kg). The surgical group included 112 patients (74 males, 38 females), ranging in age from 2 months and 6 days to 13 years and 11 months, with the youngest being 2 months and 7 days old (6 kg). The patients in the transcatheter group were older and heavier than those in the surgical group were (*p* < 0.01) (Table 1).

**Table 1:** Baseline characteristics and operative outcomes of patients.

Characteristics	Transcatheter (*n* = 39)	Surgical (*n* = 112)	*p*-value
Female (%)	26 (66.7%)	74 (66.1%)	<0.01
Age (year)	6.1 (2.8, 7.4)	1.3 (0.6, 2.8)	<0.01
Body weight (kg)	18.0 (14.0, 27.5)	9.0 (7.0, 13.0)	<0.01
BSA (m^2^)	0.7 (0.6, 1.1)	0.4 (0.3, 0.6)	<0.01
VSD size by echocardiography (mm)	5.0 (3.9, 6.0)	7.4 (5.8, 8.6)	<0.01
LVED (mm)	38.0 (34.5, 41.0)	34.0 (30.0, 38.0)	<0.01
Hospital stay (days)	5 (5, 6)	9 (8, 12)	<0.01
Ambulate postoperatively (days)	1 (0, 1)	5 (4, 6)	<0.01
Operating time (h)	1.3 (0.8, 1.8)	2.5 (2.2, 2.8)	0.443
Mechanical ventilation (days)	0	1.0 (0.4, 1.0)	<0.01
Patients retained in ICU (*n*, %)	0	3 (2.7%)	<0.01
ICU stay (h)	0	92.3 (67.0, 118.4)	<0.01
Bleeding amount (ml)	0 (0, 1)	20 (15, 30)	<0.01
Blood transfusion volume (ml)	0	100 (0, 180)	<0.01
Albumin usage (g)	0	15 (5, 20)	<0.01
New postoperative pulmonary infection (*n*, %)	1 (2.6%)	47 (42.0%)	<0.01
Use of intravenous nutrition (*n*, %)	0	87 (77.7%)	<0.01
Therapeutic use of antibiotics (*n*, %)	0	34 (30.4%)	<0.01
Fever within 72 h after operation (*n*, %)	0	88 (78.6%)	<0.01
No fever	39 (100%)	24 (21.4%)	
Mild fever	0	46 (41.1%)	
Moderate fever	0	34 (30.4%)	
High fever	0	8 (7.1%)	
VSD size on angiography (mm)	3.2 ± 1.7	–	–
Fluoroscopic time (min)	15.9 (10.0, 30.5)	–	–
Cardiopulmonary bypass (min)	–	77.5 ± 27.4	–
Aortic cross clamp time (min)	–	43.5 ± 18.5	–
Pericardial drainage volume (ml)	–	165.7 ± 71.9	–
Remove pericardial drainage tube (days)	–	3.4 ± 1.2	–

LVED, left ventricular end diastolic diameter; VSD, ventricular septal defect; BSA, body surface area; ICU, intensive care unit.

### 3.2 Closure outcomes

#### 3.2.1 Success rates

Successful occlusion was defined as the use of an appropriately sized occluder selected on the basis of the dcVSD size, stable device placement without dislodgment, and confirmation of no significant residual shunting, aortic valve dysfunction, or new arrhythmias via echocardiography and angiography. The technical success rate was 94.9% (37/39). One failed case was attributed to significant residual shunting, and the other failed case was attributed to moderate aortic regurgitation. Both patients were successfully treated via unplanned surgical repair under cardiopulmonary bypass. Surgical success was defined as complete closure of the defect during surgery, no residual shunting on echocardiography, and stable vital signs without reliance on high-dose vasopressors. The technical success rate was 100% (112/112).

#### 3.2.2 Aortic valve changes

**Figure 4:**
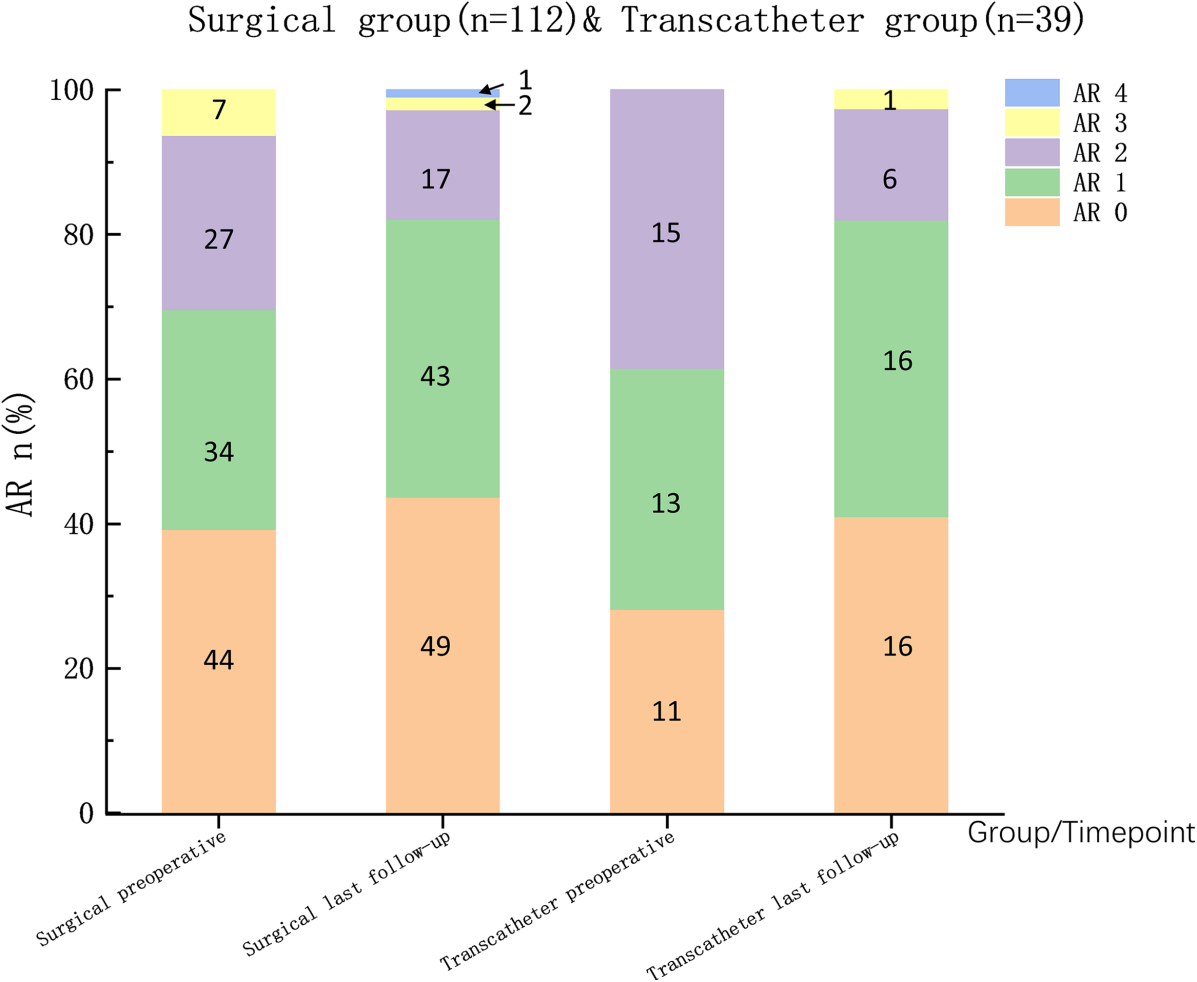
The grading of the severity of AR is based mainly on the maximal distance reached by regurgitant blood-flow images (RFIs) in the left ventricular outflow tract (12). AR 0/1/2/3/4: None/RFIs was located at aortic valve level/RFIs was between the region just inferior to the aortic valve and the middle of the level of the anterior mitral leaflet (AML)/RFIs reached tip of AML.

Preoperatively, 115 patients (76.2%) presented with mild aortic valve prolapse (AVP), which improved in both groups postoperatively. Among the 96 patients (63.6%) with preoperative aortic regurgitation (AR), 83 patients (55.0%) experienced no significant changes, 49 patients (32.5%) experienced improvement, 17 patients developed new AR, and 2 patients experienced worsening AR. No significant differences were observed between the groups in terms of postoperative aortic valve changes (Figure 4). Follow-up echocardiography confirmed that the size and function of the left ventricle were normal in patients with new or worsened AR.

#### 3.2.3 New-onset arrhythmias

Three patients (7.7%) in the transcatheter group developed new arrhythmias: incomplete right bundle branch block, occasional premature ventricular beats, and complete left bundle branch block. In all patients, normal sinus rhythm returned before discharge following conservative dexamethasone treatment. Eighteen patients (16.1%) in the surgical group experienced new arrhythmias, and two patients presented with persistent right bundle branch block at the last follow-up. Normal sinus rhythm returned in the remaining patients (Table 2).

**Table 2:** Postoperative cardiac electrophysiology and costs.

New-onset arrhythmia/cost	Surgical (*n* = 112)	Transcatheter (*n* = 39)	*p*-value
New-onset arrhythmia	18 (11.9%)	3 (2.0%)	
RBBB	7	0	
First-degree AV block	4	0	
APBs	2	0	
SVT	1	0	
IRBBB	3	1	
VPBs	1	1	
LBBB	0	1	
Total cost (Yuan)	32,002 (26,023–33,798)	48,296 (43,196–54,342)	<0.01
Treatment (Yuan)	22,554 (16,658–24,302)	28,225 (22,767–32,810)	<0.01
Comprehensive (Yuan)	9,539 (8,929–10,647)	20,294 (16,873–24,117)	<0.01

Total costs include treatment costs and comprehensive medical costs. Treatment costs include anesthesia, surgery and consumables, medications, blood and blood products, the remaining costs are categorized as comprehensive costs. RBBB, complete right bundle branch block; AV block, atrioventricular block; APBs, atrial premature beats; SVT, supraventricular tachycardia; IRBBB, incomplete right bundle branch block; VPBs, ventricular premature beats; LBBB, complete left bundle branch block.

#### 3.2.4 Residual shunting

In the transcatheter group, 5 patients (12.8%) experienced minimal residual shunting postoperatively, which resolved within three months after the start of the follow-up period. Twelve patients (10.7%) in the surgical group exhibited residual shunting: 10 minimal, 1 mild, and 1 moderate. By six months after surgery, all but one case had resolved. The patient with the moderate case underwent a second surgery to close the shunt defect, and, as the procedure was successful, there was no shunt at the ventricular level postoperatively, deterioration of the aortic valve, enlargement of the heart, or other conditions (Table 3).

**Table 3:** Post-operative residual shunt.

Groups	1 week post-operation	1 month post-operation	3 months post-operation	6 months post-operation
Transcatheter (*n* = 39)	5 (3.3%)	4 (2.6%)	0	0
	Trivial (5)	Trivial (4)		
Surgical (*n* = 112)	12 (7.9%)	3 (2.0%)	2 (1.3%)	0
	Trivial (10)	Trivia l (2)	Trivial (1)	
	Mild (1)	Moderate (1)	Moderate (1)	
	Moderate (1)			
Total (number)	17	7	2	0

Trivial residual shunt: Slight unbundled left-to-right shunt. Mild residual shunt: Residual shunt diameter is less than 2 mm. Moderate residual shunt: Residual shunt diameter is between 2 and 5 mm.

#### 3.2.5 Other postoperative complications

No serious complications, such as displacement or dislocation of the blocker, cardiac or vascular perforation, death, or hemolysis, occurred in the transcatheter group. In the surgical group, there was one case of recurrent moderate to high fever on the fifth postoperative day, and cardiac ultrasound suggested that multiple new redundancies were added to the mitral valve, which was considered new-onset infective endocarditis. After conservative treatment with antibiotics, a follow-up ultrasound on day 19 revealed regression of the growths, and no complications, such as acute heart failure, arrhythmia, or abscess fistula formation, were observed. This patient experienced preoperative shortness of breath and dysplasia of the posterior mitral valve combined with mitral valve prolapse and moderate regurgitation, which may have been the underlying disease factor that triggered infective endocarditis.

The median follow-up time was 14 (range 3–47) months in the transcatheter group and 11 (range 3–54) months in the surgical group. None of the patients had more than mild pulmonary regurgitation during the preoperative evaluation or postoperative follow-up. Right outflow tract obstruction and right ventricular enlargement were not observed in the surgical group, despite the use of the transverse transpulmonary arteriotomy route in all patients.

#### 3.2.6 Perioperative management parameters

Two transcatheter patients were hospitalized for over 6 days after surgery. One hundred surgical patients (90%) took a long time to transition to level II care and could not walk on operation day, the first postoperative day, or the eighth postoperative day. The two groups showed significant differences (all *P* < 0.01) in terms of the hospital stay, postoperative time to ambulate independently, operating time, mechanical ventilation, amount of intraoperative bleeding, amount of blood and blood products transfused intraoperatively and postoperatively, amount of albumin used, fever in the first 72 h of the postoperative period, postoperative development of new lung infections, use of intravenous nutrition, and use of therapeutic-use antibiotics (Table 1). The surgical group had three patients (2.7%) in the intensive care unit, while the transcatheter group had none. The two groups were not significantly different (*P* = 0.443 > 0.05). Intraoperative transfusion was the transfusion of exogenous blood or blood products (excluding autologous blood recycling or processing), and 81 surgical patients (72.3%) received blood products, mostly suspended erythrocytes, fresh frozen plasma, cold precipitation, and platelets. 87 surgery patients (77.7%) got intravenous nourishment. Intravenous feeding was not administered in the transcatheter group. New postoperative lung infections occurred in 47 surgical patients (42.0%) and 1 transcatheter patient (2.6%). The surgical group habitually used second-generation cephalosporin for infection prophylaxis, 34 patients (30.4%) used antibiotics therapeutically, and the transcatheter group used none. The pericardial drain was removed 3.41 ± 1.23 days and on the 4th postoperative day in 103 patients (92%) in the surgical group (Table 1). Table 4 exhibit transcatheter group and ADO-II manufacturer sheath sizes and information.

**Table 4:** Ocluder and seath.

Ocluder and seath	Manufacturer/size (F)	Number (%)	Size mm (number)
Manufacturers of occlude (ADO-II)	AGA Medical Corporation	1 (2.6%)	6 (1)
	Beijing Starway Medical Technology Inc.	38 (97.4%)	6 (4)
			7 (5)
			8 (9)
			9 (6)
			10 (10)
			11 (2)
			12 (2)
Seath size	5F	15 (38.5%)	–
	6F	17 (43.9%)	–
	7F	7 (17.9%)	–

#### 3.2.7 Inpatient costs

Significant differences in total costs, treatment costs, and medical service costs were observed between the two groups (all *p* < 0.01). Detailed cost breakdowns are provided in Table 2.

## 4 Discussion

DcVSD, caused by underdevelopment of the conal septum, results in a lack of structural support for the aortic root, with the superior margin of the defect being almost contiguous with the aortic valve root. Owing to the Venturi effect, the defect predisposes the aortic valve root to be drawn into the defect. As the condition progresses, aortic valve prolapse (AVP) may gradually worsen. Although this may reduce the left-to-right shunt volume, secondary aortic valve disease progresses gradually, particularly when the defect is large, making this impact more significant. However, a consensus has not been reached regarding whether the presence and severity of AVP or aortic regurgitation (AR) should serve as indications for surgical closure of the dcVSD. Some researchers (13–15) suggest that even mild AR warrants consideration for surgical intervention once it is detected. Furthermore, studies have shown a strong association between aortic valve disease and dcVSDs larger than 5 mm, suggesting the need for early closure of such defects (16). Other researchers advocate early intervention, regardless of the shunt volume, to slow the progression of aortic valve disease (17), while some suggest immediate surgical intervention upon the detection of AVP (16, 18). Preoperative AVP or AR—or the simultaneous presence of both—are significant risk factors for postoperative AR progression, highlighting the critical importance of early surgery in preventing aortic valve complications (19, 20).

VSD closure is one of our main concerns, with an emphasis on recently developed postoperative arrhythmias. The dcVSD, as a “high-position” defect, is anatomically advantageous due to its distance from the cardiac conduction system. Moreover, the ADO-II device successfully closes defects not via compressive force from its double-disc design but rather via expansion of its waist, which minimizes its impact on the valve and reduces pressure on the conduction system during implantation, thus lowering the risk of sustained conduction system injury. In this study, three patients in the intervention group developed new arrhythmias postoperatively. One patient experienced complete left bundle branch block on the second postoperative day, but all patients exhibited return of normal sinus rhythm after conservative treatment with dexamethasone infusion, and no new arrhythmias were observed during the midterm follow-up. Postoperative arrhythmias in both groups were transient and likely related to myocardial swelling caused by occluder release, but overall, the procedure was safe.

Another major concern, particularly in dcVSD, is the appearance of new-onest AR or a worsening of pre-existing AR. Some researchers (2, 21) have found that even after defect closure, simple patch repair for dcVSDs often fails to prevent the progression of AR, especially when preoperative AVP or AR is present. de Leval et al. (22) reported that approximately 14% of dcVSD patients who underwent patch repair required subsequent aortic valve replacement. Wang et al. (10) reported acceptable short-term outcomes in children who underwent ADO-II closure for small dcVSDs (<5 mm diameter) and only one case of new AR. However, the long-term impact on the aortic valve requires further investigation. In this study, acceptable outcomes were achieved for most patients with preoperative AR or AVP, regardless of whether ADO-II closure or patch repair was performed. Mild preoperative AVP was common in both groups and improved to some degree postoperatively. Although the midterm follow-up revealed some cases of AR progression or new onset, its severity, defined by different echocardiographic indices (23), was mild, suggesting that further intervention was not required. No ventricular dilation or other complications were observed at the final follow-up.

Additionally, in this study, one patient in the intervention group presented with signs of AR progression from mild to mild-to-moderate at the one-year follow-up. Given the timing of the worsening, it is unlikely that the occluder began affecting the valve six months postoperatively. A more probable cause is residual shunting within six months of surgery, which adversely impacts the valve, leading to the natural progression of aortic valve disease.

The findings of this study differ from those reported by Liao et al. (24), who noted that the expenses for surgical repair were 11.4% lower than those for device closure. This discrepancy may be related to the manufacturer of the occluders used. Most patients received domestic occluders. Although the costs of occluders exceed those of traditional open surgery, longer hospital stays and higher rates of postoperative complications following surgical repair result in higher overall expenses. Overall, interventional therapy is relatively inexpensive, particularly for Chongqing patients under 15 years of age, who benefit from local medical reimbursement policies, with out-of-pocket expenses less than 10,000 RMB post-reimbursement. Even for patients in developing countries, high-value surgical interventions can be performed at relatively low costs.

In this study, percutaneous closure was performed successfully even in pediatric patients with mild AVP or AR, with no significant postoperative impact on the aortic or pulmonary valves. Although closure is recommended for dcVSDs smaller than 5 mm in current research (10), this study included 11 patients with defects larger than 5 mm. With sufficient technical support and appropriate patient selection, percutaneous closure may be attempted for defects up to 10 mm in size in patients weighing over 10 kg, even in the presence of mild AVP or AR. For such cases, percutaneous closure may be a less invasive and more cost-effective option. However, for patients with other cardiac anomalies requiring surgical intervention, open surgery remains preferable to avoid multiple procedures. Although the mortality rate is low, careful consideration of surgical risks and their impact on the aortic valve is necessary.

For reference, our team has compiled some of the key experiences from the dcVSD interventional occlusion method for reference. Initially, Selecting the appropriate angiographic angle is vital for accurately assessing the defect size and location. On the basis of our experience, the optimal angle for dcVSDs is a left anterior oblique projection at 90° with a 20° cranial tilt. If the details of the defect remain unclear, adjustments to a right oblique at 30° with a 20° cranial tilt can provide higher quality images and ensure procedural success. Second, due to the defect's close proximity to the valve, it is very challenging to guide the wire from the left heart system through the defect into the right heart system to establish a track. To facilitate wire passage, a portion of the angiographic catheter can be trimmed. Additionally, because of the unique anatomical location of the defect, advancing the delivery sheath into the left ventricle can also be difficult. In this case, we recommend using a sheath with a smaller diameter first and ensuring that the sheath tip is positioned as high as possible, which makes it relatively easier to advance the sheath into the left ventricle. Finally, during occluder deployment, positioning the waist of the occluder at the defect site and extending slightly more of the occluder into the left ventricle can help minimize the occluder's impact on the aorta. In addition to the above recommendations, we still advise that comprehensive hemodynamic assessment using Doppler color flow and angiographic techniques is recommended to evaluate the VSD size and shunting preoperatively. Accurate defect measurement and appropriate occluder sizing are critical for reducing the surgical duration, the risk of residual leakage, and the risk of occluder displacement. If percutaneous closure is unsuccessful, patients can be quickly transitioned to open surgical repair under cardiopulmonary bypass via an intraoperative “green channel” to ensure safety and optimal outcomes.

## 5 Advantages and limitations

This study has several advantages. First, data used in this study were obtained from a large pediatric medical center in Western China, providing results with a certain degree of representativeness. A significant number of dcVSD cases were included, allowing for a more robust analysis. Finally, this is the first study to compare interventional occlusion and surgical repair specifically for dcVSD closure, offering valuable insights into the relative benefits of each approach. This study has several limitations. First, As this study was a single-center retrospective analysis with a restricted sample size and probable selection bias, the results need to be regarded with caution, and multicenter, larger-scale, and long-term follow-up studies are needed to confirm the efficacy and safety of ADO-II occlusion. Second, the occluder was selected on the basis of previous reports and the surgeon's experience, and standardized protocols are lacking. This introduces potential confounding factors related to differences in devices. Finally, the intervention group consisted of older and heavier children than the surgical group did, reflecting the real-world patient population at the study center. This is attributable to the requirements of interventional procedures, which depend on adequate vascular conditions for occluder delivery. The use of the ADO-II device is less restricted by age and weight because of its smaller delivery sheath, which lacks internal filling material. Occlusion was successful in patients under 10 kg, but such cases require a highly skilled surgeon. In future studies, researchers should adjust for age and weight to derive more rigorous conclusions.

## 6 Conclusion

This study revealed that ADO-II occlusion for pediatric dcVSD achieves high success rates with acceptable follow-up outcomes and no severe complications. Compared with open surgical repair under cardiopulmonary bypass, percutaneous occlusion is minimally invasive, inexpensive, allows for a faster recovery, avoidance of cardiopulmonary bypass and blood transfusions, no significant progression of AR or need for surgical intervention during short- to mid-term follow-up.

For patients without significant AVP, with mild AR, a body weight >10 kg, and pulmonary hypertension or no other associated cardiac anomalies that require surgical treatment, percutaneous intervention can be considered the first-line treatment. However, given the potential long-term impact of occluders on the aortic valve and conduction system, extended follow-up is essential.

## Data Availability

The raw data supporting the conclusions of this article will be made available by the authors, without undue reservation.
